# Charge density waves in disordered media circumventing the Imry-Ma argument

**DOI:** 10.1038/srep31897

**Published:** 2016-08-24

**Authors:** Hitesh J. Changlani, Norm M. Tubman, Taylor L. Hughes

**Affiliations:** 1 Department of Physics, University of Illinois at Urbana-Champaign, Urbana, Illinois 61801, USA; 2 Department of Chemistry, University of California, Berkeley, Berkeley, California 94720, USA

## Abstract

Two powerful theoretical predictions, Anderson localization and the Imry-Ma argument, impose significant restrictions on the phases of matter that can exist in the presence of even the smallest amount of disorder in one-dimensional systems. These predictions forbid electrically conducting states and ordered states respectively. It was thus remarkable that a mechanism to circumvent Anderson localization relying on the presence of *correlated* disorder was found, that is also realized in certain biomolecular systems. In a similar manner, we show that the Imry-Ma argument can be circumvented, resulting in the formation of stable ordered states with discrete broken symmetries in disordered one dimensional systems. We then investigate other mechanisms by which disorder can destroy an ordered state.

Disorder can have drastic effects on electronic properties, especially in low dimensions. On the one hand, it lifts the degeneracy between competing phases through “order by disorder” mechanisms^1^,^2^,^3^, and on the other it localizes clean metallic states^4^,^5^,^6^,^7^,^8^, and even creates unusual emergent excitations^9^,^10^. In one dimension, the essential physics of disorder is captured by Anderson localization^4^ for transport properties, and work related to the seminal paper of Imry and Ma^11^,^12^,^13^ for understanding the disorder-driven destruction of ordered phases. The dimensionality dependence of both effects weakens with increasing spatial dimension, thus their strongest effects are seen in one dimension.

An interesting exception to Anderson localization arises due to *correlations* in the disorder. In particular, refs. 14 and 15 discovered a class of non-interacting random *n*-mer models where a band of single-particle states which exhibit no backscattering exist; a condition key for circumventing localization^16^. In all *n*-mer models there are two types of ‘atoms’, which we call *A* and *B*, differing only in their on-site energy, which are placed at random on a one-dimensional chain; with the condition that *n B*’s (an “*n*-mer”) are always placed consecutively. With the inclusion of nearest neighbor repulsive interactions, the Hamiltonian for spinless electrons that we study is,


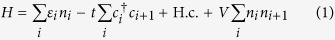


where 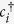 (*c*_*i*_) and *n*_*i*_ refer to the usual spinless electron creation (destruction) and density operators respectively on site *i* which is occupied by either an *A* or *B* atom, and correspondingly the on-site energy 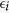 is either 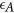 (set to zero throughout) or 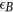, which will be referred to as the “disorder strength”, *t* is the nearest-neighbor hopping parameter, and *V* is the nearest-neighbor interaction strength. We use the terminology “monomer”, “dimer”, “trimer”, and “quadrumer” for the cases *n* = 1, 2, 3, 4 respectively. We also note that the random dimer model has been of particular interest due to its possible relevance to understanding transport in biomolecules^17^,^18^,^19^,^20^,^21^.

The disorder-free system at small *V*/*t* is known to be a Tomonaga-Luttinger liquid^22^ which at half-filling, and a critical interaction strength (*V*/*t* = 2), forms a charge density wave (CDW) state that remains stable for all larger *V*/*t*^22^,^23^. However, according to the Imry-Ma argument^11^,^24^, such a state should not exist upon the slightest introduction of disorder; the focus of this article will be to show how the *n*–mer models avoid this.

Given a pinned, commensurate CDW with every *even* site (mostly) occupied, as is depicted in Fig. 1, let us assess its stability to the introduction of weak disorder (for which we closely follow ref. 24). Consider a segment of length 2*L* that is part of a 1D lattice with a large number of sites, and divide it into odd and even sublattices, to be labelled as 1 and 2 respectively. If the sum of all the on-site energies on the even sites is greater than the sum of the on-site energies on all the odd sites, then it is energetically favorable for each electron in the segment to shift by one site, despite the cost of the repulsive interaction of neighboring electrons, hence forming a domain wall.

For *uncorrelated* disorder, and for *L* sufficiently large to apply statistical arguments, the difference between the summed energies on the two sublattices is of the order 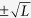. Since forming a domain wall costs only an energy of order *V*, the former effect always wins for some large enough *L*; hence, the system acts to *reduce* its energy by the formation of domains. Thus, there is no (quasi) long-range CDW order in one dimension upon the slightest introduction of *uncorrelated* disorder.

However, the situation is markedly different when the disorder is *correlated*. Let us define *n*_*α*,*j*_ to be the number of sites where *α* is an index for the disorder site (*A* or *B*), and *j* is the sublattice index (1 or 2). Then, for *any* disorder realization of the random dimer model, and *any* interval of 2*L* sites, we have the conditions,


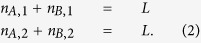


Since the instances of *B* occur *only* as dimers, *n*_*B*, 1_ *must* be equal to *n*_*B*, 2_ (assuming the segment of length 2*L* does not contain any incomplete dimers) which, in turn, implies that *n*_*A*, 1_ = *n*_*A*, 2_, i.e., the number of *A*-type sites on each sublattice are also exactly equal.

We emphasize that the relationship (2) holds globally and, more importantly, locally for any subset with an even number of lattice sites. Thus, the difference between summed on-site energies on the even and odd sublattices is *zero*, i.e.,


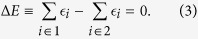


This energy difference does not grow with *L*, and therefore the Imry-Ma argument for the formation of domains is not expected to apply. In fact, the condition *n*_*B*, 1_ = *n*_*B*, 2_, and hence Eq. (3), holds for any *n*-mer model with *n* even. This is demonstrated in Fig. 2 which shows the special cancellation (or lack thereof) of the sublattice energy imbalance for the even (odd) *n*–mer models for an ensemble of disorder realizations. In instances of segments where one or more boundary cuts a dimer in half, there is an edge correction of one or two lattice sites, which is small on the scale of *L* and does not affect our conclusions in the regime of weak-to-moderate disorder (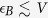).

We verify these arguments by performing numerically accurate density matrix renormalization group (DMRG)^25^ calculations of the *n*–mer models for *n* = 1, 2, 3, 4, discussed further in the Methods section. Results from our simulations for individual disorder realizations are shown in Fig. 3 where we have plotted the electronic density on every *even* site for *V* = 5*t* and three disorder strengths. The boundary conditions have been chosen to slightly favor the high occupation of the *even* sites, and thus any rapid decrease from high to low density is the signature of a domain wall.

At 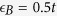, the random monomer and trimer models show large but finite domains whose size decreases with increasing disorder strength. In comparison, the random dimer and quadrumer show no tendency to form domain walls up to a critical (*V*-dependent) disorder strength. For example, for all of the individual random dimer and quadrumer realizations in Fig. 3, the first domain walls are seen only around 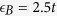 when *V* = 5*t*.

The eventual occurrence of domain walls in the random dimer and quadrumer models can be explained as follows. First, for sufficiently large disorder 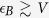, the effect of the heretofore ignored edges in the *n*-mer version of the Imry-Ma argument now starts to play an important role. The energy of the CDW is now reduced by order 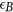, which is greater than the price of forming a domain wall (order *V*). Second, any *B* site would like to have lower density wherever possible, causing fluctuations of the density that grow large enough to destroy the ordered state. For example, for the realization in Fig. 3, for 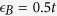, the density fluctuations are seen to be small (~0.03), compared to the maximum occupation of a site (~0.95), and eventually grow past 0.5, at which point CDW order is lost. To further understand the mechanisms for domain creation, additional studies were performed and are presented as part of the Supplemental Information.

Let us now look beyond individual realizations and perform statistical analyses of our samples; Fig. 4 shows the average size of the CDW domains as a function of disorder strength. As is anticipated from the Imry-Ma argument, the random monomer and trimer models show divergence in domain size around *vanishing* disorder for all *V*/*t* considered. This is in contrast to the random dimer and quadrumer models which have no domain walls until a *critical* 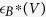 is reached.

In conclusion, we have explored an aspect of the interplay between interactions and disorder in one dimensional systems, an exciting avenue for both theory and experiments. Using an interacting version of *n*–mer models where Anderson localization is avoided, we have explicitly shown that the Imry-Ma argument for destroying CDW order does not directly hold either, for even *n*. In the absence of a sub-dominant mechanism that destroys the order at small disorder strength, charge density waves are stabilized in media with correlated disorder. From the experimental viewpoint, of particular relevance are recent cold atom studies that have created and measured the strength of charge density waves in one dimensional geometries in the presence of quasiperiodic (correlated) disorder^26^; similar setups for spinless fermions could provide the first controlled test of the existence of the phenomenon proposed here.

## Methods

### Numerical techniques

“Our simulations of disordered fermions were performed with the DMRG^25^ algorithm” i.e. please eliminate “density matrix renormalization group” as the acronym DMRG has been defined earlier. Also note change of tense “are performed” is the wrong tense and has been changed to “were performed”. The DMRG technique is based on the principle that a many-body wavefunction can be written as a matrix product state (MPS) whose parameters (elements of the matrices) can be efficiently optimized. The accuracy of the MPS representation for describing the exact state is dependent on the dimension of the matrices (denoted as the bond dimension *m*). While any wavefunction is exactly representable as an MPS in the *m* → ∞ limit, the utility of the DMRG technique/MPS decomposition is apparent when *m* is small (typically a few hundred to a few thousand). This is especially true for the low energy states of gapped one dimensional systems with short-range hoppings and interactions, with open or pinned boundary conditions. The DMRG calculations in this paper were performed with a combination of our codes, the open-source ITensor software^27^, and the Algorithms and Libraries for Physics Simulations (ALPS)^28^ library.

We work with spinless electrons at half filling on chains with an even number of sites. At zero disorder and large *V*/*t*, for a chain with open boundary conditions, it is favorable for CDW order to develop and spread from both ends. These CDW orders emerge from opposite sublattices and hence interfere in the bulk of the chain; such a superposition of states can restore translational invariance. While the existence of the underlying CDW order can still be detected in the bulk from density-density correlations, it is easier (and computationally more efficient) to explicitly break the symmetry between the two orders. This allows us to access the CDW order directly by simply measuring electron *density* at every site.

To break the sublattice symmetry, we apply a pinning field in all cases (with or without disorder) to the left-most site, by using an on-site energy 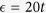 at that site. No pinning field is applied at the right end. This suppresses the electron occupation at the left-most site to a low value and ensures that the CDW emerging from the left boundary is in phase with the CDW that naturally emerges from the right boundary. This choice of boundary conditions induces only a small bias towards a stable CDW. This is verified in our findings showing that the random monomer and trimer models do not have stable CDW phases even when the disorder is small, and that the random dimer and quadrumer models transition into the disordered phase at a sufficiently large interaction dependent disorder strength.

Such symmetry breaking strategies also reduce the entanglement of the ground state quantum many-body wavefunction, allowing for smaller bond dimensions to be used in the DMRG algorithm. For our calculations *m* = 300–500 was used, which yielded truncation errors of the order of 10^−14^ to 10^−8^, with variations for different realizations and disorder and interaction strengths.

### Disorder averaging and measurements

**Figure 4:**
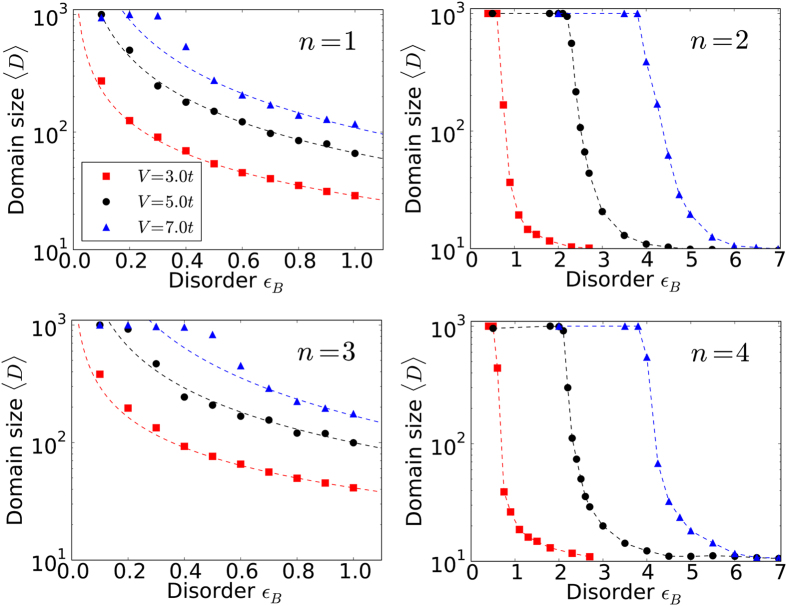
Domain size versus disorder. Profiles of the disorder-averaged domain size (〈*D*〉) versus disorder-strength (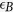 in units of *t*) for the spinless fermion random monomer (*n* = 1), dimer (*n* = 2), trimer (*n* = 3) and quadrumer (*n* = 4) models at half filling at various interaction strengths *V*. Around 80 disorder realizations, each of 1000 sites, were used for the averaging procedure. The dashed lines indicate approximate trends and serve as guides to the eye. The critical disorder strength for the occurrence of finite domains in the odd *n*–mer cases is consistent with zero in concordance with the Imry-Ma argument. In the even *n*–mer case, the critical disorder strength is non-zero.

In Fig. 4 we presented results for disorder-averaged domain sizes as a function of the disorder strength, using about 80 realizations each of length 1000 sites. A “domain” was defined as a region where local CDW (on either sublattice) was present. This was identified by monitoring “domain walls” - regions where the (dominant) occupation of the electrons changed from one sublattice to the other.

This was done, in practice, by traveling along the chain and identifying regions where the electronic occupation of the dominant sublattice slipped to a value below 0.5. There is a small error (of the order of 10 sites or less) in recording the domain size, introduced due to the domain wall being an extended object. Each disorder realization generically yields multiple domains, the typical size (and hence number per sample) being disorder and interaction strength dependent. While correlations in the location of domain walls in a single realization should exist, here we have ignored these and simply considered each domain as a statistically independent sample for the averaging procedure. Thus the disorder averaging is done over multiple realizations and domain sizes within each realization.

## Additional Information

**How to cite this article**: Changlani, H. J. *et al*. Charge density waves in disordered media circumventing the Imry-Ma argument. *Sci. Rep.* **6**, 31897; doi: 10.1038/srep31897 (2016).

## Supplementary Material

Supplementary Information

## Figures and Tables

**Figure 1:**
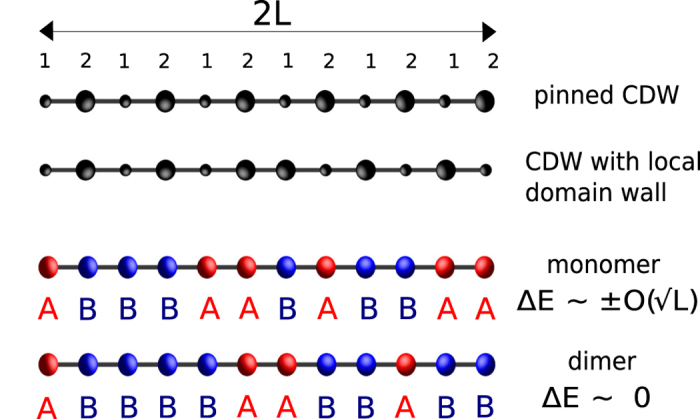
Charge density waves on a disordered lattice. The top panel shows a schematic of the electronic density (proportional to area of circles) demonstrating formation of domain walls in a charge density wave in a generic disordered model. The Imry-Ma argument predicts that such domains are (typically) energetically favorable even for the smallest non-zero disorder. The bottom panel shows schematics of a segment of length 2*L* in the random monomer and random dimer models. The red and blue sites correspond to *A* type (monomers with on-site energy 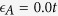) and *B* type (monomers or dimers with on-site energy 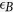) sites respectively. For the random monomer case, the typical difference in summed sublattice energies of the order of 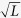 while it is zero in the random dimer case.

**Figure 2:**
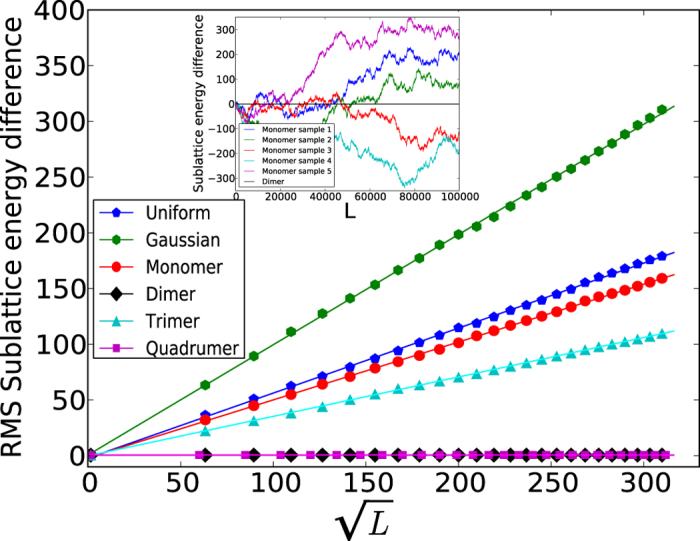
The Imry-Ma argument for different types of disorder. Root mean squared value of Δ*E* (the difference in summed sublattice energies) versus the length of the segment *L* computed for six types of disorder distributions. For each disorder type, 2000 realizations, each comprising of 10^5^ sites, were used. The uniform (box) distribution corresponds to maximum and minimum energies of 1 and −1 respectively, the Gaussian distribution has a mean of 0 and a spread (*σ*) of 1, and the *n*–mer models (*n* = 1, 2, 3, 4) each have 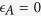 and 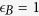. Inset: Δ*E* vs *L* for several individual disorder realizations of the random monomer and dimer models. The former shows large fluctuations in Δ*E* while the latter has Δ*E* = 0 or ±1 (not visualized on the scale of the plot).

**Figure 3:**
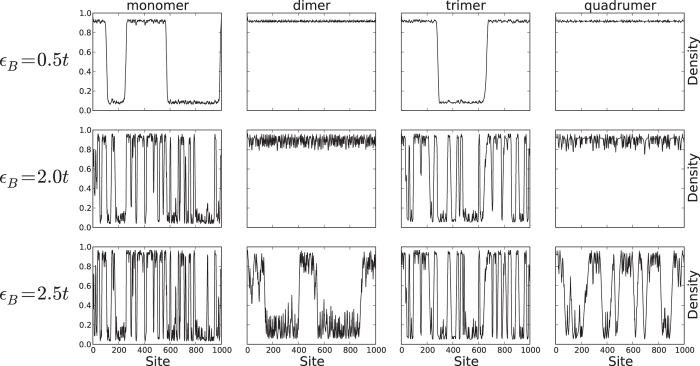
Domain wall formation in charge density waves. Fermionic density on every *even* site for individual realizations of the random monomer, dimer, trimer and quadrumer models at disorder strengths 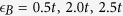 for *V* = 5*t*. At small disorder, the odd-*n* models show the formation of domain walls in agreement with the Imry-Ma argument, while the even-*n* models, which circumvent the argument, do not (within the size considered). Beyond some critical disorder strength, domain wall formation is favorable for all models, i.e., CDW order persists only in local patches.
