# Erratum: Charge density waves in disordered media circumventing the Imry-Ma argument

**DOI:** 10.1038/srep35698

**Published:** 2016-12-09

**Authors:** Hitesh J. Changlani, Norm M. Tubman, Taylor L. Hughes

Scientific Reports 6: Article number: 3189710.1038/srep31897; published online: 08 24 2016; updated: 12 09 2016

The original version of this Article contained errors in the Methods section.

““Our simulations of disordered fermions were performed with the DMRG^25^ algorithm” i.e. please eliminate “density matrix renormalization group” as the acronym DMRG has been defined earlier. Also note change of tense “are performed” is the wrong tense and has been changed to “were performed””.

now reads:

“Our simulations of disordered fermions were performed with the DMRG^25^ algorithm”.

