# Supplementary material for: Charge density waves in disordered media circumventing the Imry-Ma argument
Source: Sci Rep. 2016 Aug 24;6:31897. doi: 10.1038/srep31897 (PMC4995405; doi:10.1038/srep31897)
Supplement: Supplementary Information [file srep31897-s1.pdf]

# Supplemental Information for “Charge density waves in disordered media circumventing the Imry-Ma argument"

Hitesh J. Changlani,[1] Norm M. Tubman,[2,1] and Taylor L. Hughes[1]

[1]*Department of Physics, University of Illinois at Urbana-Champaign, Urbana, Illinois 61801, USA*

[2]*Department of Chemistry, University of California, Berkeley, California 94720, USA*

(Dated: June 19, 2016)

## I. FINITE SIZE EFFECTS

As is discussed in the main paper, density matrix renormalization group (DMRG) [1] calculations were performed for interacting spinless electrons on $L = 1000$ site chains, using 80 realizations for each type of disorder. The nearest neighbor hopping and density-density interaction strengths are $t$ (set to 1 throughout) and $V$ respectively. For this $L$, the finite size effects for all disorder types (random monomer, dimer, trimer and quadrumer) are negligible for disorder strengths ($\epsilon_B$) where the typical domain sizes are much smaller than $L$.

For example, for the random monomer case, the Imry-Ma argument [2] holds and the chains are disordered i.e. there is no long range charge density wave (CDW) order and the average domain size ($\langle D \rangle$) is finite at any finite $\epsilon_B > 0$. This is seen in Fig. S1 (left panel). The average domain sizes, computed for independent ensembles of $L = 1000$ and $L = 2000$ sites for $V/t = 5$, essentially coincide with each other at a given $\epsilon_B$. (In fact, the small difference between the ensembles is well within the error due to the finite number of realizations used in the estimation of the average.) The error bars shown in Fig. S1 correspond to the standard deviation of the distribution of domain sizes.

The finite size effects are more pronounced in the regions where an order to disorder transition occurs. This is primarily because of the contribution of domain sizes that exceed the length of the sample. (When no domain wall is found in the sample, we simply set the domain size $D$, to be used for averaging, to be $L$. This introduces a bias in the estimation of $\langle D \rangle$). Depending on the value of $\epsilon_B$ and the type of correlated disorder, such samples may have finite weight in the ensemble and thus their contribution to $\langle D \rangle$ may be significant.

This is highlighted in Fig. S1 (right panel) which shows the difference between the $L = 1000$ and $L = 2000$ site ensembles of the random dimer model near the critical disorder strength ($\epsilon_B^*$)

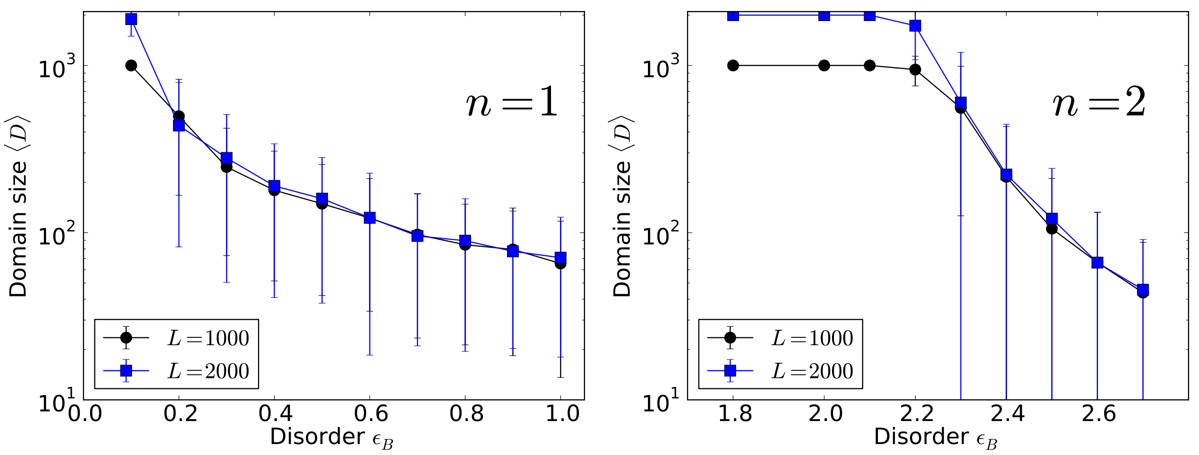


Figure S1. (Color online): Finite size dependence of the average domain size ($\langle D \rangle$) as a function of disorder strength ($\epsilon_B$ in units of the hopping $t$) at $V/t = 5$ for the random monomer ($n = 1$, left panel) and dimer ($n = 2$, right panel) models using ensembles of $L = 1000$ and $L = 2000$ site chains. The error bars denote the standard deviation of the distribution ($\sigma$); for most points the error on the mean ($\sigma/\sqrt{N_{samples}}$) is of the order of the size of the symbols, or smaller.

i.e. the disorder strength where the first finite domains begin to form. Since the finite size effects are large in the vicinity of this point, precisely estimating $\epsilon_B^*$ would need a scaling theory, which remains to be developed for the form of correlated disorder considered here. However, a rough estimate of $\epsilon_B^*$ can still be made to within $\sim \pm 0.5t$.

## II. RESULTS FOR SPECIAL INSTANCES OF DISORDERED CHAINS

We now describe two special instances of disorder realizations that help understand the mechanisms of formation of domain walls (or phase slips). They are illustrated in Fig. S2.

### A. Single dimer in a clean chain

Consider a chain of even length $L$, with sites numbered from $1, 2...L$. Then place a dimer at the center of the chain i.e. at sites $i = L/2$ and $i = L/2 + 1$ set $\epsilon_i = \epsilon_B$, and for the rest of the chain $\epsilon_i = \epsilon_A = 0.0t$ (except the left boundary which has the pinning field described in the Methods section of the main paper). This chain is schematically depicted in Fig. S2 (top panel).

We monitor the CDW order in the sample as a function of $\epsilon_B$. For this, we plot the charge

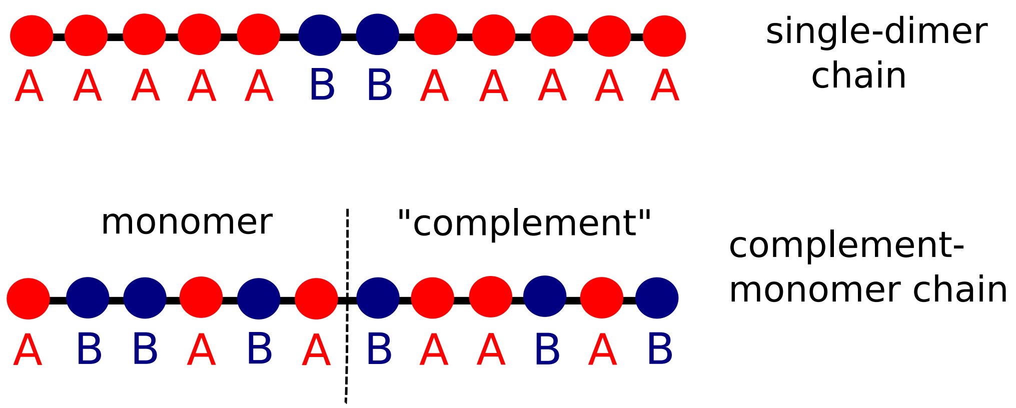


Figure S2. (Color online): Special one dimensional chains for demonstrating the mechanism of domain wall formation. Top: A single dimer of $B$ sites is embedded in an otherwise clean chain made of $A$ sites. Bottom: The complement-monomer chain is constructed by first generating an instance of a random monomer of length $L/2 = 6$ sites. Then its "complement" is defined as the chain obtained by replacing every $A$ by $B$ and $B$ by $A$. The monomer and complement are joined end to end to form the length $L = 12$ chain.

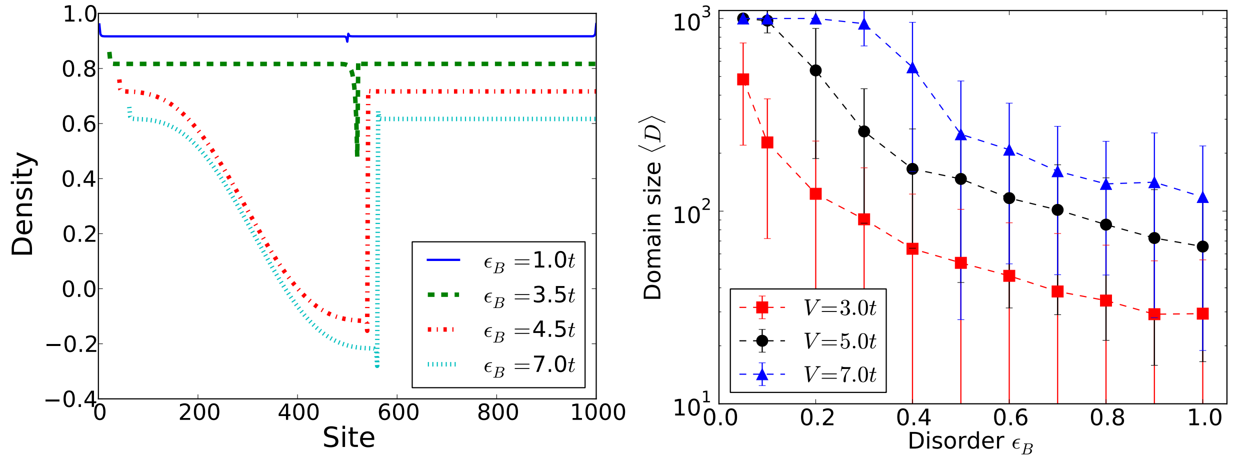


Figure S3. (Color online): Left: Electron density on even sites for the chain with a single dimer embedded in the center, for representative disorder potential strengths on the dimer $\epsilon_B$, at $V = 5.0t$. The density is shifted by amounts $(0.00, 0.10.0.20, 0.30)$ for $\epsilon_B/t = (1.0, 3.5, 4.5, 7)$ and correspondingly the location of the dimer (x axis) is shifted by amounts $(0, 20, 40, 60)$. Right: Average of domain wall size ($\langle D \rangle$) versus $\epsilon_B$ for the "complement" monomer chain for various $V/t$. The error bars denote the standard deviation of the distribution.

density only on even sites, any transitions from high to low occupations in this plot indicate a domain wall i.e. phase slip of the CDW order. As is seen in Fig. S3 (left panel), for small $\epsilon_B$ the CDW order persists; a small kink at the central sites is seen, indicating the slightly lower occupation of the $B$ site compared to the other high occupation $A$ sites participating in the CDW. This mild suppression of the electron density allows the CDW to have the same phase on both sides of the chain. However, at a critical $V$-dependent disorder strength, it is unfavorable for either of the $B$ sites to be (mostly) occupied. Once the occupation on this dimer is forced to be low, the $A$ sites on either end of it acquire high occupation numbers. Since these two $A$ sites are on opposite sublattices, the CDW on either side of the dimer is out of phase.

Despite this phase slip at the center of the sample, the pinning field at the left end eventually forces the CDW to change phase, gradually over the length of half the sample. This is the origin of the asymmetry (about the center of the chain) seen in the charge density in Fig. S3 (left panel).

### B. "Complement"-monomer chain

In the main paper we noted that the Imry-Ma argument is circumvented when the sublattice energies satisfy the condition,

$$\sum_{i\in 1}\epsilon_i - \sum_{j\in 2}\epsilon_j = 0 \tag{1}$$

where $\epsilon_{i(j)}$ refers to on-site energies on sites $i(j)$, and $1, 2$ is used to refer to sublattice indices. We emphasized that this condition must hold at all short and long length scales.

To provide an explicit example where this condition holds globally but not locally, we define the "complement" chain. This is constructed by generating a random monomer chain of $L/2$ sites and its "complement"; the latter obtained by replacing every $A$ type site by a $B$ type site and vice versa. Then these two segments are placed such that the end of the monomer connects to start of the complement, to form a $L$-site chain. A special instance of this chain is schematically depicted in Fig. S2 (bottom panel). We generated about $50$ such disorder realizations and studied the formation of domains on varying the disorder strength $\epsilon_B$.

As is shown in Fig. S3 (right panel), we find that for any non-zero disorder, $\langle D\rangle$ is finite (i.e. domain walls form), consistent with the Imry-Ma argument. While this is empirically evident for $V/t = 3$, there is a plateau-like feature for $V/t = 5$ and $7$ at small disorder, similar to the one observed in the odd $n-$mer case (see Fig. 4 of the main text). This is attributed to the finite size of the lattice ($L = 1000$ sites) which cannot support domains bigger than this scale. (However, we

also note that based only on the data presented we can not completely rule out the possibility that the complement-monomer chain orders at a non-zero $\epsilon_B$ for some sufficiently large $V/t$.)

[1] S. R. White, Phys. Rev. Lett. **69**, 2863 (1992).

[2] Y. Imry and S.-k. Ma, Phys. Rev. Lett. **35**, 1399 (1975).
